# Influence of Genotype, Maturity Stage and Year on Surface Pitting Susceptibility and Related Physiological Traits in Sweet Cherry (*Prunus avium* L.)

**DOI:** 10.3390/plants15010063

**Published:** 2025-12-25

**Authors:** Pavol Suran, Veronika Danková, Aneta Bílková, Lucie Plecitá

**Affiliations:** Vyzkumny a Slechtitelsky Ustav Ovocnarsky Holovousy Ltd., Holovousy 129, 50801 Hořice v Podkrkonoší, Czech Republic

**Keywords:** anthocyanins, antioxidant capacity, ascorbic acid, dry matter, polyphenols, *Prunus avium*, surface pitting

## Abstract

This study investigates the resistance of sweet cherry (*Prunus avium* L.) accessions to surface pitting, a critical postharvest issue. A two-year analysis of 32 accessions and 3 cultivars, fruit chemical composition (total antioxidant capacity, anthocyanins, polyphenols, dry matter, and ascorbic acid), and maturity stages on pitting susceptibility was conducted. The damage index (DI) is a quantitative measure of fruit susceptibility to surface pitting after mechanical injury and storage; lower DI values indicate higher resistance, while higher DI values indicate greater susceptibility. The damage index (DI) in our study ranged from 1.18 to 2.87, with ‘10072’ exhibiting the highest resistance (DI = 1.18 ± 0.20) and ‘16806’ the lowest (DI = 2.87 ± 0.49). Biochemical analysis showed an inverse correlation between total dry matter content (TDM) and DI (r = −0.445, *p* < 0.001), with high-TDM accessions demonstrating lower pitting susceptibility. Ascorbic acid levels also negatively correlated with DI (r = −0.148, *p* < 0.01), as ‘10072’ contained 41% higher ascorbic acid than ‘16806’. In contrast, total antioxidant capacity (TEAC) correlated positively with DI (r = 0.309, *p* < 0.001), suggesting that higher antioxidant levels may increase susceptibility. Maturity stages affected fruit chemical composition but did not significantly alter DI values (first maturity stage DI = 2.17 ± 0.56; second maturity stage DI = 2.19 ± 0.53, *p* > 0.05). Some accessions maintained stable resistance across seasons, emphasizing the role of genetics. These findings provide valuable insights for breeding strategies to improve cherry resistance to surface pitting. The identification of highly resistant accessions like ‘10072’ offers promising candidates for breeding programs, while predictive chemical markers could aid rapid selection for enhanced postharvest quality.

## 1. Introduction

Sweet cherries (*Prunus avium* L.) are among the most economically significant fruit crops due to their exceptional flavor, nutritional value, and consumer demand. However, their marketability and postharvest quality are often compromised by surface pitting—a physiological disorder characterized by small depressions on the fruit surface. This issue drastically reduces the shelf life and market value of cherries, with studies reporting that up to 80% of transported cherries are affected by pitting, surpassing other common postharvest disorders such as decay caused by *Penicillium* [1,2].

Surface pitting arises from a complex interplay of factors. Mechanical damage during harvesting, transportation, and handling is a major contributor, compromising the integrity of the fruit’s skin and accelerating tissue breakdown [3,4,5]. Such damage creates microcracks and ruptures in the skin, triggering physiological and biochemical changes that increase the risk of surface pitting during storage [6]. Additionally, these damaged areas act as entry points for pathogens, leading to rapid decay and mold growth [7]. At the basic level, surface pitting is associated with a reduced cellular turgor pressure, cell wall damage, and enzymatic activity. Turgor loss causes shrinkage of cells and the formation of cavities in between them, while damage to the middle lamella facilitates the activity of enzymes such as pectin methylesterase (PME) and polygalacturonase (PG), which accelerate cell wall degradation [8,9,10,11,12]. This breakdown is further exacerbated by changes in membrane permeability, disrupted water homeostasis, and increased ethylene production following physical damage [13,14,15,16].

Genetic predisposition also plays a critical role in determining resistance to surface pitting. Cultivars with thicker skins or higher levels of structural polysaccharides are generally more resistant to mechanical damage. For example, studies have shown that cultivars such as ‘Bing’ and ‘Regina’ exhibit lower susceptibility to pitting compared to thinner-skinned cultivars like ‘Sweetheart’. Recent molecular analyses have further identified differences in gene expression related to cell wall metabolism and both the expression of antioxidant enzymes and genes within pathways responsible for the production of small-molecule antioxidants as key factors influencing resistance [17,18,19].

The biochemical composition of cherries has also been linked to their susceptibility to surface pitting. Polyphenols, anthocyanins, and ascorbic acid are among the compounds most frequently studied for their roles in mitigating oxidative stress and preserving cell wall integrity. While higher levels of polyphenols are generally associated with enhanced antioxidant capacity, some studies suggest that excessive accumulation of the easily extractable polyphenol fraction may weaken cell walls by altering their structural properties [20,21,22]. Conversely, ascorbic acid has been shown to stabilize cellular membranes and reduce oxidative damage, potentially enhancing resistance to mechanical stress [22,23].

Pre- and post-harvest treatments have been explored as strategies to mitigate surface pitting. Pre-harvest applications of calcium salts or growth regulators have been reported to enhance fruit firmness and reduce susceptibility to mechanical damage [20]. Post-harvest interventions such as modified atmosphere packaging (MAP), UV-C irradiation, and antioxidant treatments have also shown promise in preserving fruit quality by reducing oxidative stress and maintaining cell wall integrity during storage [21,22,23,24,25,26]. However, the efficacy of these treatments varies depending on factors such as genotype, harvest timing, and storage conditions.

Despite these advances, significant gaps remain in understanding how genetic variability interacts with biochemical composition and environmental factors to influence resistance to surface pitting. Previous studies have often focused on a limited number of cultivars or experimental conditions, leaving many questions unanswered regarding the broader applicability of their findings.

This study aims to address these gaps by conducting a comprehensive two-year evaluation of 35 sweet cherry accessions from diverse genetic backgrounds. The primary objectives are to assess the influence of genotype on resistance to surface pitting; investigate the relationships between fruit chemical composition—including total polyphenols, anthocyanins, total antioxidant capacity (TEAC), dry matter content, and ascorbic acid—and resistance to surface pitting; and evaluate the impact of maturity stages on both physiological traits and susceptibility to surface pitting.

By integrating genetic variability with detailed physiological analyses, this research seeks to provide actionable insights for breeding programs aimed at developing sweet cherry cultivars with improved postharvest durability.

## 2. Results

### 2.1. The Influence of Type of Accession on Damage Index

**Figure 1:**
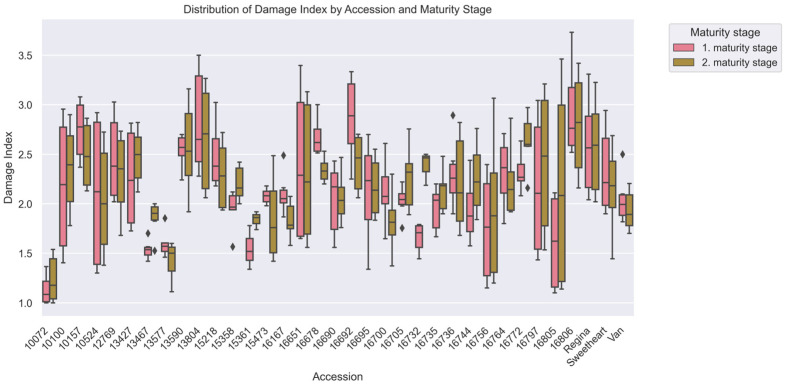
Comparative evaluation of surface pitting resistance among cherry (*Prunus avium* L.) accessions using nonparametric statistical analysis (Kruskal–Wallis H test; *p* < 0.05). Individual cherry accessions (x-axis) are ranked by their damage index scores for surface pitting (y-axis). The box plot was generated from data collected on 150 fruits per accession. Each box represents an accession and provides an overview of the variability in resistance index values within that specific accession. Box sections display the middle 50% of data (IQR) between Q1 and Q3, with the median shown as a horizontal line through the box center. Range Visualization: Whiskers extend outward from Q1 to the lowest non-outlier value and from Q3 to the highest non-outlier value, typically encompassing values within 1.5 times the IQR. Discrete data points extending beyond the whisker margins denote outliers where the deviation from either lower or upper quartile surpasses 1.5× the interquartile range, signifying anomalous or exceptionally divergent values. Longer boxes and whiskers indicate greater variability in the damage index values for a given accession, whereas shorter boxes and whiskers suggest lower variability and thus higher data homogeneity.

The damage index (DI), evaluated across 35 sweet cherry (*Prunus avium* L.) accessions over two years, demonstrated significant genotypic variability, with values ranging from 1.18 to 2.87. Lower damage index values indicate higher resistance to surface pitting, making this parameter critical for assessing fruit quality during storage and handling. Accession ‘10072’ consistently exhibited the highest resistance, with an average damage index of 1.18 (±0.20), significantly outperforming standard cultivars such as ‘Van’ (1.99 ± 0.22) and ‘Regina’ (2.58 ± 0.48). In contrast, accession ‘16806’ displayed the lowest resistance, with an average index of 2.87 (±0.49), highlighting its susceptibility to mechanical damage (Figure 1).

Yearly trends revealed that certain accessions maintained consistent performance across growing seasons, as evidenced by low variability in their resistance indices. Accession ‘10072’, for instance, exhibited an average damage index of 1.18 (±0.20) over two years, with no statistically significant differences (*p* > 0.05) between years, underscoring its genetic stability. In contrast, accession ‘13577’ showed minor fluctuations in its damage index across years, ranging from 1.53 (±0.06) to 1.48 (±0.23) (*p* > 0.05). Conversely, accessions such as ‘13804’ exhibited greater variability, with resistance indices ranging from 2.28 (±0.15) to 3.21 (±0.24), reflecting a stronger influence of environmental factors.

The mean damage index for all accessions was 2.17 (±0.56) during the first maturity stage, whereas the second maturity stage exhibited a very similar average of 2.19 (±0.53) (Table 1). The small numerical increase observed was not statistically significant (*p* > 0.05). Accession ‘13577’, notably, sustained comparable performance during both maturity stages, with damage index of 1.60 (±0.13) throughout the first maturity stage and 1.76 (±0.33) at the time of the second maturity stage (Figure 1). Conversely, accession ‘16805’ exhibited a rise in its damage index during the second maturity stage (2.17 ± 0.96), indicating decreased resilience under later maturity stage conditions.

### 2.2. The Influence of Fruit Chemical Composition on Damage Index

Analysis of 35 sweet cherry accessions demonstrated a significant positive correlation between total antioxidant capacity (TEAC) and damage index (DI) (r = 0.309, *p* < 0.001) (Table 2). This relationship remained consistent across both study years. Accessions with elevated TEAC levels (≥120 µmol Trolox/100 g) exhibited higher susceptibility to surface pitting compared to low-TEAC accessions (≤80 µmol Trolox/100 g). For instance, accession ‘15218’ (127.21 ± 17.51 µmol Trolox/100 g) displayed an DI of 2.39, whereas low-TEAC accession ‘15361’ (41.53 ± 1.95 µmol Trolox/100 g) showed a significantly lower DI of 1.69. TEAC exhibited significant negative correlations with total anthocyanins (r = −0.323, *p* < 0.001) and dry matter content (r = −0.483, *p* < 0.001). No meaningful associations were observed between TEAC and polyphenol content (r = 0.019, *p* > 0.05) or ascorbic acid levels (r = −0.058, *p* > 0.05).

Total dry matter (TDM) emerged as a critical determinant of surface pitting resilience, spanning 9.28–18.64% across accessions. A notable negative correlation was detected between TDM and DI (r = −0.445, *p* < 0.001). High-TDM accessions such as ‘16797’ (18.68 ± 2.74% TDM) exhibited 26% higher DI compared to low-TDM accession ‘15361’ (DI 1.69; 14.37 ± 0.35% TDM). Fruits from second maturity stage demonstrated synergistic interactions, with TDM increasing by 12.3% alongside a 13.7% rise in ascorbic acid levels. TDM showed moderate positive correlations with ascorbic acid (r = 0.260, *p* < 0.001), anthocyanins (r = 0.554, *p* < 0.001), and polyphenols (r = 0.316, *p* < 0.001).

Total anthocyanin content displayed a weak negative association with damage index (r = −0.187, *p* < 0.05). Polyphenol content showed a weak negative relationship with surface pitting susceptibility (r = −0.086, *p* > 0.05), despite maintaining notable positive relationships with anthocyanins (r = 0.389, *p* < 0.001) and dry matter (r = 0.316, *p* < 0.001).

Ascorbic acid levels negatively correlated with damage index (r = −0.148, *p* < 0.001), with high-ascorbic acid accessions demonstrating lower pitting susceptibility compared to low-content accessions. Positive relationships were identified between ascorbic acid and dry matter (r = 0.260, *p* < 0.001) as well as anthocyanins (r = 0.228, *p* < 0.001).

Multivariate analysis revealed complex interactions among compositional variables. The negative correlation between TEAC and dry matter (r = −0.483, *p* < 0.001) contrasted with the positive anthocyanin-TDM relationship (r = 0.554, *p* < 0.001). Fruits from second maturity stage exhibited coordinated increases in TDM and ascorbic acid.

### 2.3. Maturity Stage Dynamics

Analysis of maturity stages revealed no significant variation in TEAC values (*p* > 0.05), with fruits of first maturity stage averaging 106.70 ± 25.57 μmol Trolox/100 g (range: 39.32–144.41) compared to 105.88 ± 31.09 μmol Trolox/100 g (37.95–144.25) in the second maturity stage (Table 1). Accession ‘10072’ exhibited exceptional stability across maturity stages (111.01 ± 13.37 vs. 111.38 ± 14.75 μmol Trolox/100 g), while ‘16167’ showed extreme fluctuation (72.70 ± 31.56 to 120.68 ± 4.67 μmol Trolox/100 g). A majority of accessions (51.4%, *n* = 18) demonstrated higher TEAC during the first harvest, with accession ‘16797’ showing a 34.6% reduction between maturity stages (114.13 ± 1.76 vs. 74.61 ± 1.37 μmol TE/100 g).

Fruits of second maturity stage contained 144% higher anthocyanin concentrations (9.48 ± 11.16 mg/100 g) compared to fruits of first maturity stage (3.88 ± 5.04 mg/100 g, *p* < 0.001) (Table 1). Accession ‘13467’ displayed a 24-fold increase (0.75 ± 0.68 vs. 18.19 ± 13.84 mg/100 g), contrasting with ‘16797’, which maintained stable levels (1.92 ± 0.00 vs. 1.71 ± 0.78 mg/100 g).

Fruits of second maturity stage showed 12.4% higher TDM (13.73 ± 3.00%) fruits of first maturity stage (12.23 ± 2.59%, *p* < 0.001) (Table 1). Accession ‘13804’ exhibited the most pronounced increase (11.94 ± 3.26% to 15.62 ± 4.71%), while ‘16772’ displayed minimal variation (±0.22% between maturity stages). The commercial cultivar ‘Regina’ maintained consistently high TDM across both maturity stages (14.18 ± 1.95% vs. 14.61 ± 1.92%).

Bidirectional trends emerged in polyphenol content, with fruits of first maturity stage averaging 28.93 ± 14.83 mg GAE/100 g vs. 29.49 ± 11.29 mg GAE/100 g in fruits of second maturity stage (*p* > 0.05) (Table 1). Of the cohort, 65.7% (*n* = 23) of accessions showed 43.8% polyphenol increases in the second harvest, while 37.1% (*n* = 13) exhibited 29.7% reductions. Accession ‘12769’ demonstrated a sharp decline (−62.5%, 53.27 ± 14.01 to 19.95 ± 3.94 mg GAE/100 g), whereas ‘16772’ increased by 33.1% (25.11 ± 3.60 to 33.43 ± 1.13 mg GAE/100 g). Stable performers included ‘16805’ (16.00 ± 5.93 vs. 16.47 ± 1.61 mg GAE/100 g) and ‘10072’ (22.49 ± 2.79 vs. 21.57 ± 1.59 mg GAE/100 g), the latter showing only 4.1% variation.

Fruits of second maturity stage contained 13.7% higher ascorbic acid (6.87 ± 2.10 mg/100 g) than fruits of first maturity stage (6.04 ± 1.85 mg/100 g, *p* < 0.001) (Table 1). Accession ‘16167’ showed a 50% increase (6.53 ± 1.32 vs. 9.80 ± 0.32 mg/100 g), while ‘16705’ exhibited similar gains (6.53 ± 1.32 vs. 9.80 ± 0.32 mg/100 g). The commercial cultivar ‘Sweetheart’ maintained stable levels (4.60 ± 2.40 vs. 4.82 ± 1.76 mg/100 g).

### 2.4. Genotypic Variability

**Figure 2:**
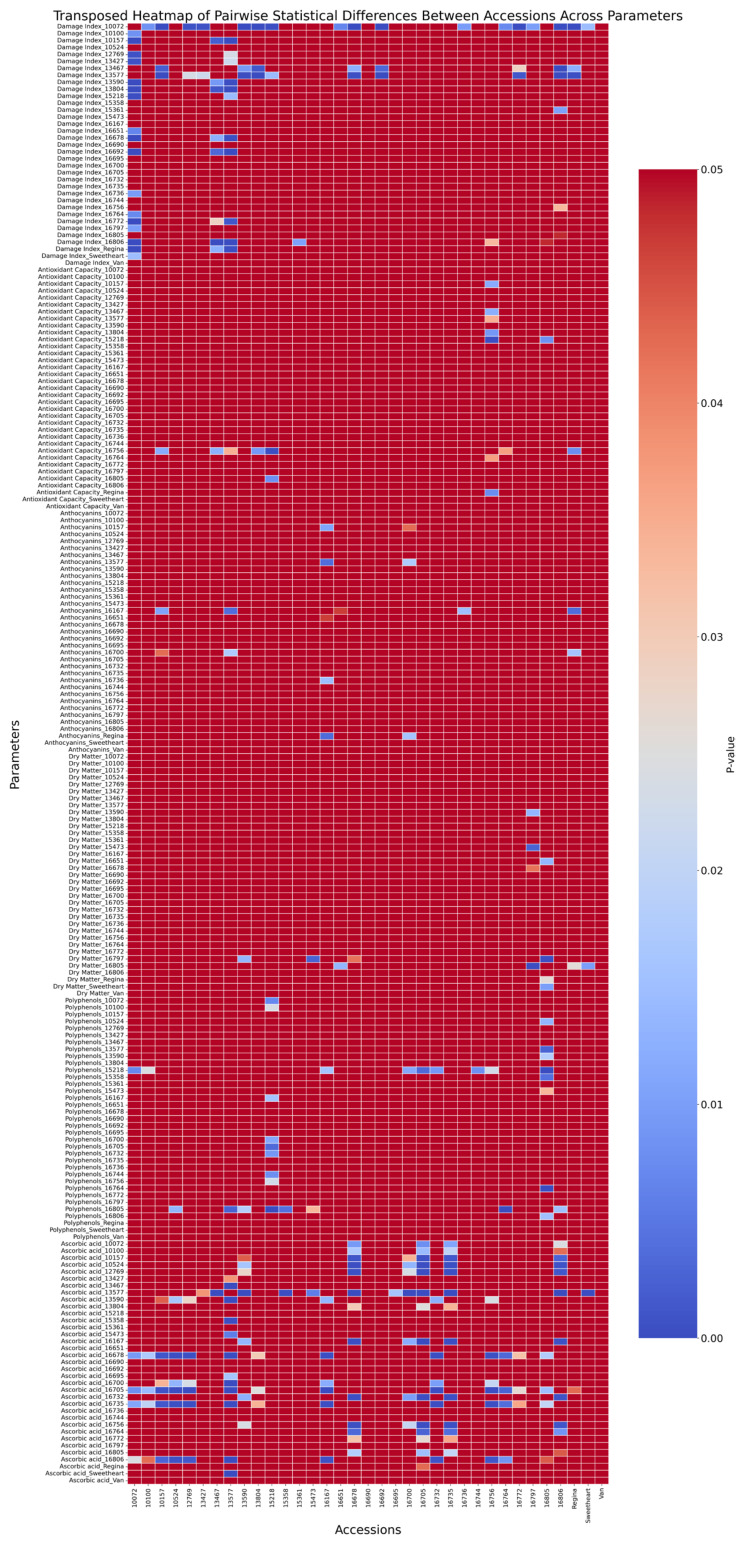
Visualization in the form of a heatmap of pairwise *p*-value differences between cherry (*Prunus avium* L.) accessions across several parameters at the second maturity stage. The x-axis corresponds to accessions, whereas the y-axis includes parameters like the Damage Index, Antioxidant Capacity, Anthocyanins, Dry Matter, Polyphenols, and Ascorbic Acid. Color gradation indicates the *p*-value (0–0.05), with darker blue signaling more significant statistical differences (*p* closer to 0) and red indicating weaker significance (*p* closer to 0.05). Non-significant comparisons are represented by uniform red shading.

**Figure 3:**
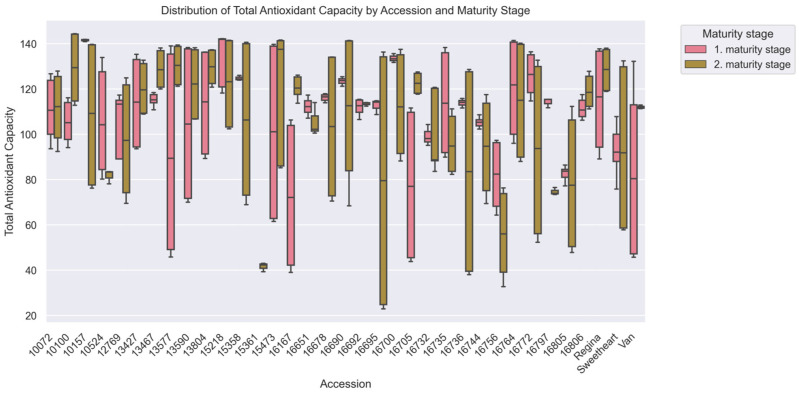
Total Antioxidant Capacity (µmol Trolox/100 g) among cherry (*Prunus avium* L.) accessions at two maturity stages, evaluated via Kruskal–Wallis ANOVA (*p* < 0.05). The x-axis corresponds to accessions, and the y-axis to Total Antioxidant Capacity values. Boxplots are colored by harvest, with pink for the 1st maturity stage and green for the 2nd, showing median, IQR, and within-accession variability for each maturity stage.

Kruskal–Wallis analysis revealed critical genotypic differences (H = 87.83, *p* < 0.001), with post hoc tests identifying 8 distinct accession pairs (Figure 2). Susceptible accession ‘Regina’ exhibited higher TEAC (121.73 ± 18.89 μmol Trolox/100 g) than resistant ‘10072’ (111.19 ± 14.71 μmol Trolox/100 g) (Figure 3), reinforcing the counterintuitive link between elevated antioxidant capacity and pitting vulnerability. Extreme contrasts emerged between ‘15218’ (127.21 ± 17.51 μmol Trolox/100 g) and ‘15361’ (41.54 ± 1.95 μmol Trolox/100 g), representing a 3.0-fold difference in TEAC corresponding to divergent damage index.

**Figure 4:**
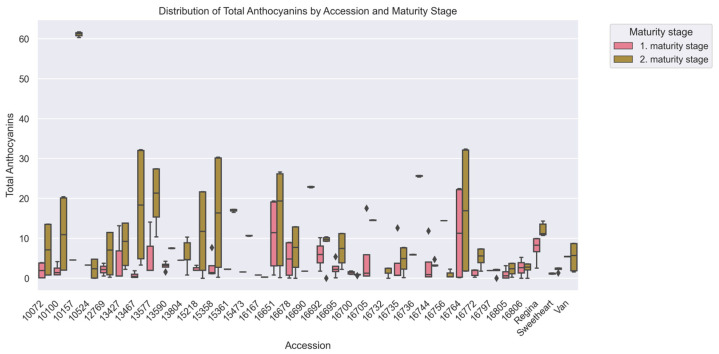
Total Anthocyanins (mg/100 g) among cherry (*Prunus avium* L.) accessions at two maturity stages, evaluated via Kruskal–Wallis ANOVA (*p* < 0.05). The x-axis corresponds to accessions, and the y-axis to Total Anthocyanins values. Boxplots are colored by harvest, with pink for the 1st maturity stage and green for the 2nd, showing median, IQR, and within-accession variability for each maturity stage.

Substantial genetic divergence (H = 85.42, *p* < 0.001) was observed, with 8 accession pairs differing significantly (Figure 2). The commercial cultivar ‘Regina’ contained 15 times higher anthocyanins (9.85 ± 3.29 mg/100 g) than ‘16167’ (0.63 ± 0.27 mg/100 g, *p* < 0.01) (Figure 4).

**Figure 5:**
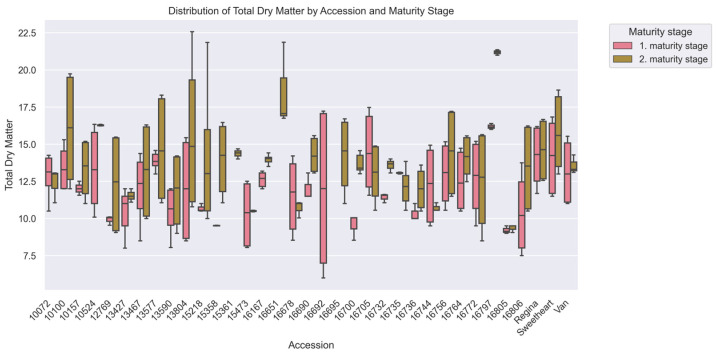
Total Dry Matter (%) among cherry (*Prunus avium* L.) accessions at two maturity stages, evaluated via Kruskal–Wallis ANOVA (*p* < 0.05). The x-axis corresponds to accessions, and the y-axis to Total Dry Matter values. Boxplots are colored by harvest, with pink for the 1st maturity stage and green for the 2nd, showing median, IQR, and within-accession variability for each maturity stage.

Extreme genotypic divergence (H = 89.08, *p* < 0.001) was confirmed, with 7 accession pairs showing significant differences (Figure 2). Resistant accession 16805 (DI 1.88; 9.28 ± 0.24% TDM) exhibited 55.17% lower TDM than susceptible ‘Regina’ (DI 2.58; 14.40 ± 2.03% TDM, *p* < 0.05) and 61.1% lower than ‘Sweetheart’ (DI 2.24; 14.95 ± 2.70% TDM *p* < 0.01) (Figure 5). Intermediate TDM accessions like ‘10072’ (DI 1.18; 12.71 ± 1.31%) demonstrated an optimal balance between structural integrity and physiological protection.

**Figure 6:**
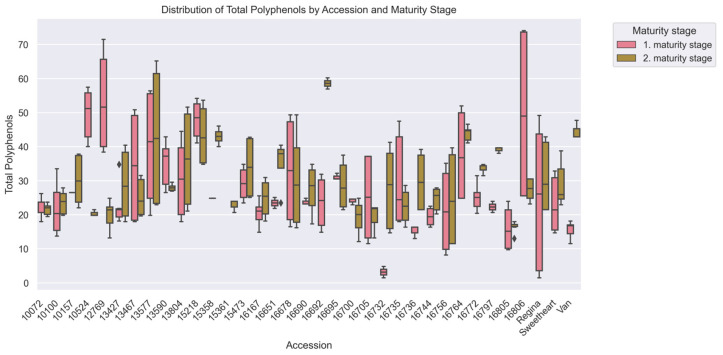
Total Polyphenols (mg GAE/100 g) among cherry (*Prunus avium* L.) accessions at two maturity stages, evaluated via Kruskal–Wallis ANOVA (*p* < 0.05). The x-axis corresponds to accessions, and the y-axis to Total Polyphenols values. Boxplots are colored by harvest, with pink for the 1st maturity stage and green for the 2nd, showing median, IQR, and within-accession variability for each maturity stage.

Significant genetic divergence (H = 44.11, *p* < 0.001) was observed, with 16 accession pairs differing markedly (Figure 2). Susceptible ‘15218’ (DI 2.38; 45.67 ± 7.57 mg GAE/100 g) contained 2.8 times higher polyphenols than resistant ‘16805’ (DI 1.88; 16.23 ± 4.54 mg GAE/100 g, *p* < 0.01) (Figure 6), despite a weak overall correlation with DI (r = −0.086, *p* > 0.05).

**Figure 7:**
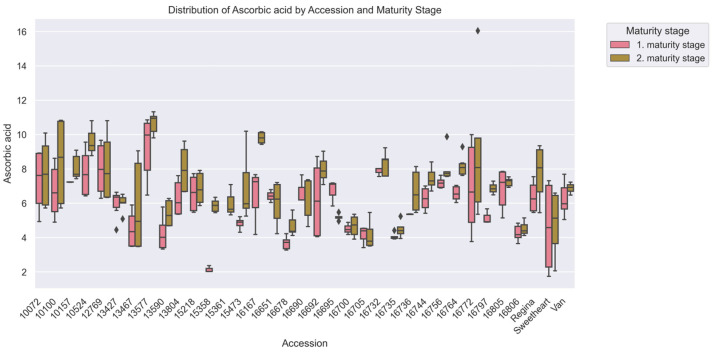
Ascorbic Acid (mg/100 g) among cherry (*Prunus avium* L.) accessions at two maturity stages, evaluated via Kruskal–Wallis ANOVA (*p* < 0.05). The x-axis corresponds to accessions, and the y-axis to Ascorbic Acid values. Boxplots are colored by harvest, with pink for the 1st maturity stage and green for the 2nd, showing median, IQR, and within-accession variability for each maturity stage.

Analysis confirmed significant divergence (H = 44.11, *p* < 0.001), with 71 accession pairs differing substantially (Figure 2). Resistant ‘10072’ (DI 1.18; 7.52 ± 1.75 mg/100 g) contained 41% higher ascorbic acid than susceptible 16806 (DI 2.87; 4.39 ± 0.39 mg/100 g, *p* < 0.001) (Figure 7). Intermediate accessions, including ‘Van’ (DI 1.99; 6.45 ± 0.87 mg/100 g), showed comparable levels.

### 2.5. Interannual Stability

TEAC levels exhibited moderate interannual variation, with 2021 values 39% higher than 2020 (88.92 vs. 123.92 µmol Trolox/100 g, *p* < 0.001) (Table 3). Accession ‘16756’ demonstrated exceptional stability across years (variation: ±3.99 µmol Trolox/100 g), while ‘16736’ showed the largest fluctuation (±81.86 µmol Trolox/100 g).

Environmental factors significantly influenced anthocyanin accumulation, with 2020 levels 5 times higher than 2021 (12.00 ± 11.34 vs. 2.45 ± 3.25 mg/100 g, *p* < 0.001) (Table 3). Accession ‘16797’ maintained exceptional stability (±0.32 mg/100 g), contrasting with ‘16764’, which exhibited substantial interannual variation (±26.11 mg/100 g).

TDM levels in 2020 were 41% higher than 2021 (15.52 ± 2.08% vs. 10.99 ± 1.61%, *p* < 0.001) (Table 3). Accession ‘16167’ showed minimal variation (2020: 12.62 ± 0.48%; 2021: 13.97 ± 0.29%), while ‘13804’ fluctuated markedly between years (11.94 ± 3.26% to 15.62 ± 4.71%). The commercial cultivar ‘Regina’ exhibited intermediate stability, with a 3.0% interannual difference.

Mean polyphenol content decreased by 34.3% between 2020 (33.37 ± 11.84 mg GAE/100 g) and 2021 (24.85 ± 12.98 mg GAE/100 g, *p* < 0.001) (Table 3). Accession ‘16744’ displayed exceptional consistency (±0.77 mg GAE/100 g), whereas ‘13577’ showed a 59.4% reduction (58.85 ± 4.01 to 23.88 ± 3.07 mg GAE/100 g). Commercial cultivar ‘Regina’ exhibited moderate variability (±30.13 mg GAE/100 g annual change).

Ascorbic acid levels varied by 5% between years (2020: 6.62 ± 1.95 mg/100 g; 2021: 6.30 ± 2.07 mg/100 g, *p* > 0.05) (Table 3). Accession ‘16744’ remained stable (±0.04 mg/100 g), while ‘16772’ fluctuated 28.5% (±5.35 mg/100 g). The commercial cultivar ‘Van’ showed intermediate variability (±10% annual change).

## 3. Discussion

The damage index (DI) analysis of 35 sweet cherry (*Prunus avium* L.) accessions over two years highlights the significant genotypic variability in fruit susceptibility to surface pitting [18]. This variability is influenced by both genetic, environmental factors, and physiological factors (ripening time, developmental stage), as genetic composition affects cell wall integrity and metabolite production, while environmental factors such as temperature and rainfall influence fruit firmness and physiological composition. Fruit at advanced ripeness stages exhibits lower susceptibility to surface pitting, with ripening progression regulating polysaccharide composition and metabolic changes that influence cell wall strength. [18]. Accessions ‘10072’ and ‘13577’ demonstrated the highest resistance, while accession ‘16806’ was the most susceptible, emphasizing the importance of genetic selection in improving postharvest fruit quality [27,28]. These findings are consistent with previous studies reporting cultivar-dependent resistance to surface pitting [2].

Environmental stability observed in certain accessions suggests strong genetic control over resistance traits, while accessions with fluctuating DI values, such as ‘13804’, indicate environmental modulation. Firmness and cell wall structure play key roles in resistance. A higher proportion of alcohol-insoluble residues (AIRs) enhances mechanical strength, while increased pectin solubilization weakens tissue resilience [18]. Variations in temperature, rainfall, or harvest maturity likely explain year-to-year fluctuations, as lower temperatures tend to increase mechanical susceptibility [29].

The correlation between fruit chemical composition and DI elucidates the mechanisms underlying resistance. The moderate positive correlation between total antioxidant capacity (TEAC) and DI (r = 0.309, *p* < 0.001) suggests that antioxidant equilibrium influences cell wall stability [30]. Although this result contrasts with some prior findings, it can be explained by genotype-specific antioxidant pathways and enzyme activity, particularly of pectin methylesterase and polygalacturonase [6,17,30]. Most antioxidant data represent soluble fractions, but phenolic compounds also exist in bound forms that contribute to total antioxidant activity [31]. The higher ascorbic acid content of resistant accession ‘10072’ compared to ‘16806’ (7.52 vs. 4.39 mg/100 g) supports its possible contribution to maintaining structural integrity [32].

A significant negative correlation between total dry matter (TDM) and DI (r = −0.445, *p* < 0.001) underscores the importance of tissue density and mechanical strength in pitting resistance [17]. TDM is a reliable indicator of firmness and resilience, confirming its role as a key determinant of postharvest quality [2].

Maturity stages significantly influenced DI variability. Fruits from later maturity stages were slightly more prone to surface pitting, aligning with previous observations that delayed harvest reduces firmness through cell wall degradation and increased anthocyanin accumulation [33,34,35,36,37]. The observed 12.4% increase in TDM in later maturity stages indicates a partial compensatory effect but not complete mitigation. Environmental factors such as temperature and precipitation affect dry matter accumulation, sugar content, and fruit texture [38,39,40].

Kruskal–Wallis analysis revealed clear genetic clustering of accessions, supporting the existence of genotype-specific resistance mechanisms. Structural attributes such as cuticle thickness, epidermal density, and cell wall elasticity influence mechanical behavior [18,19]. Cultivars with compact epidermal layers and thicker cuticles are generally more resistant to pitting due to improved stress distribution and cell cohesion [14,18]. Genotypic differences in enzyme activity, particularly pectin methylesterase and polygalacturonase, may further explain variability in resistance [17].

Climatic variability between years strongly affected physiological traits. TEAC remained moderately stable, whereas anthocyanin content fluctuated markedly, indicating environmental sensitivity of secondary metabolism [29]. High temperatures increase sugar accumulation, while rainfall may dilute soluble solids, influencing fruit firmness and resistance [40,41]. The observed 41% reduction in TDM between years supports reports of climate-driven changes in fruit composition. Similarly, fluctuations in humidity and solar radiation influence anthocyanin biosynthesis, contributing to interannual variation in firmness and color [42]. These findings confirm the substantial environmental influence on cherry physiological properties and highlight the need for adaptive cultivation strategies.

Although total polyphenol content and TEAC values were generally stable between maturity stages (Table 1), differences in anthocyanins and dry matter suggest that multiple factors influence the antioxidant profile. The lack of a strict parallel between TEAC and total polyphenols, especially in the interannual comparison (Table 3), may reflect contributions from other antioxidants such as ascorbic acid or nitrogen- and sulphur-based compounds, and the influence of seasonal growing conditions [43,44,45]. Interestingly, accessions with higher resistance indices tended to show higher polyphenol, anthocyanin, and ascorbic acid levels, indicating that specific genotypic traits may underlie mechanical resilience even when overall seasonal trends differ [17,18].

Overall, the data reveal a multifactorial relationship between biochemical components and mechanical resistance, shaped by genetic variability. Weak correlations between DI and components such as TEAC, anthocyanins, polyphenols, and ascorbic acid highlight the complexity of these traits, suggesting indirect effects mediated through biochemical and structural interactions. This variability among accessions supports the integration of multiple physiological and biochemical indicators into breeding programs [46].

The slightly positive correlation between total antioxidant capacity (TEAC) and the damage index (r = 0.309, *p* < 0.001) contrasts with some prior findings [30], but remains consistent with the idea that oxidative status may influence enzymatic processes related to cell wall remodeling, as reported in studies linking stress-induced protein and enzyme changes with pitting susceptibility [6,17,30]. Differences among studies likely arise from cultivar variation, environmental effects, and methodological differences in TEAC determination [17]. In addition, antioxidant capacity reflects a combination of diverse compounds such as polyphenols, carotenoids, nitrogenous metabolites, sulphur compounds, and vitamins, whose proportions vary across genotypes and assays [43,44,45,47,48,49]. This compositional complexity explains inconsistent correlations across studies.

The moderate positive relationship between TEAC and resistance suggests that antioxidants may stabilize cell wall polysaccharides and limit oxidative degradation, thereby contributing indirectly to firmness [50]. TEAC exhibited negative correlations with total dry matter (r = −0.483) and anthocyanins (r = −0.323), indicating possible metabolic trade-offs between antioxidant potential and structural accumulation [51].

Anthocyanins showed moderate variation among accessions and maturity stages, with a weak negative correlation to DI (r = −0.187, *p* < 0.05). This indicates an indirect rather than direct contribution to resistance [35,52]. Their established role as antioxidants mitigating oxidative damage supports this interpretation [53]. The weak correlation of anthocyanins with the resistance index, and their link to total polyphenols (r = 0.389), indicates that polyphenols likely contribute more to fruit antioxidant capacity and resistance than anthocyanins [54]. Environmental and genotypic factors jointly regulate anthocyanin biosynthesis through light, water, and temperature responses, as well as transcription factors of the MYB and bHLH families [46,55,56,57,58]. While these mechanisms were not directly analyzed in this study, they help explain inter-accession differences in pigment and resistance levels.

Total dry matter (TDM) emerged as a robust indicator of mechanical resistance, reflecting its structural function in firmness and fruit resilience [2,17,59]. A negative correlation between TDM and TEAC suggests an inverse balance between biochemical and physical defense mechanisms [60,61]. The interaction between dry matter composition, enzymatic activity, and oxidative stability supports the idea that firmness-related traits indirectly affect antioxidant efficiency [60].

Polyphenols demonstrated dual structural and antioxidant roles. Their variability among accessions may explain differences in firmness and postharvest stability. While higher polyphenol concentrations are generally linked to antioxidant protection, certain phenolic compounds can also modify cell wall structure, potentially reducing mechanical resistance [62,63]. The Folin–Ciocalteu method used to determine total phenolics may further complicate interpretation due to cross-reactivity with other reducing agents [64]. Nonetheless, polyphenols remain essential for maintaining cell wall cross-linking and defense integrity [65,66].

Ascorbic acid showed a weak negative correlation with DI (r = −0.148, *p* < 0.01), confirming its limited direct role in mechanical resistance but highlighting its contribution to redox homeostasis [67,68]. Its indirect influence likely occurs through synergy with other antioxidants such as tocopherols and enzymatic scavengers [69,70,71]. Although ascorbic acid’s pro-oxidant behavior under metal-rich conditions may reduce its efficiency [69], maintaining balanced levels supports oxidative stability and stress resilience [72].

Future research should explore the interactions between physiological factors, such as anthocyanins, dry matter, and ascorbic acid, with structural traits like cell wall composition to better understand their impact on resistance. Investigating epigenetic factors and their role in fruit ripening could further clarify the genetic basis of resistance to surface pitting. Additionally, analyzing regulatory networks and transcription factors could provide insights for breeding programs aimed at enhancing fruit resilience.

Our study provides a comprehensive analysis of fruit chemical compounds and surface pitting incidence, considering genetic variability and multi-year observations. The findings highlight significant variations in resistance and interactions between maturity stages and fruit chemistry. Standardizing puncture tests with mechanized systems could improve accuracy in future research.

Practically, identifying resistant accessions like ‘10072’ and ‘13577’ offers valuable breeding candidates. Predictive chemical analyses, such as total dry matter and antioxidant capacity, could serve as rapid screening tools. Growers can use this information to select suitable cultivars and optimize maturity stages, improving post-harvest durability. Integrating genetic, environmental, and epigenetic insights into breeding and cultivation strategies will further enhance cherry quality and resilience.

## 4. Materials and Methods

### 4.1. Experimental Design

The study evaluated 32 cherry (*Prunus avium* L.) accessions from the Holovousy breeding program over two consecutive years (2020–2021) to account for climatic variability. Two maturity stages were considered within each harvest season. ‘Van’ was used as a control cultivar due to its documented resistance to surface pitting [2], while ‘Regina’ and ‘Sweetheart’ were selected for their known susceptibility to mechanical damage [17,18].

#### 4.1.1. Plant Material

The experiment was conducted on sweet cherry (*Prunus avium* L.) trees at VSUO Holovousy in Eastern Bohemia, Holovousy, Czech Republic (50.383629° N, 15.576902° E, 360 m above sea level). The site is classified as a moderately warm macro-region with a moderately humid sub-region, featuring a long-term mean annual temperature of 8.4 °C and an average annual precipitation of 663.5 mm. The orchard, established in 2008, consists of trees grafted on Gisela 5 rootstock, trained as free spindles, and planted at a spacing of 5 × 1.5 m. No covering systems or supplemental irrigation were used.

#### 4.1.2. Orchard Management

Standard agricultural practices for conventional sweet cherry production were applied. Trees were fertilized according to the standard orchard practice applied in the region. The spaces between rows were managed as permanent pasture, regularly mowed or mulched, whereas tree rows were kept free of weeds using herbicides. Annual pruning was performed before flowering, with the resulting biomass mulched into the inter-row spaces.

#### 4.1.3. Fruit Picking

Fruits were picked at two maturity stages evaluated through visual observation with the PNW Dark Sweet Cherry Development Index Chart (Oregon State University, Hood River, OR, USA). The first maturity stage took place three days prior to commercial maturity (shades 2–3), while the second maturity stage represented full commercial maturity (shades 4–6). A total of 50 fruits per accession were randomly picked in 3 replications and used for both surface pitting evaluation and subsequent storage experiments. Another dosage of 1200 g of fresh fruits was randomly picked for chemical compounds analysis.

#### 4.1.4. Fruit Preparation

After picking, the 50 fruits per accession described in Section 4.1.4. were selected, free of visible defects and diseases, and were initially cooled to 4 °C and 85%relative humidity for 4 h preceding start the experimental procedures [2]. All of these fruits were designated for the subsequent mechanical damage assessment described in the following section.

#### 4.1.5. Storage Condition and Induction of Mechanical Damage

Fruits were maintained at 1.5 °C for 21 days under modified atmosphere packaging (MAP) conditions (0.5–1% O_2_ and 10–40% CO_2_). MAP conditions were chosen because this is the most common method used for fruit storage and transport to the final market, ensuring conditions relevant to commercial practice. To assess mechanical damage susceptibility, a manual puncture test was conducted using a handheld digital force gauge (ZTA 20N, IMADA, Toyohashi, Japan) using a 5 mm diameter probe. The gauge was held stationary, and the right cheek of each fruit was manually pressed against the probe tip until a 0.5 N force was used. The rate of fruit application to the probe was not controlled, simulating realistic mechanical impacts that occur during handling.

Following mechanical induction, all fruits were immediately placed in MAP storage as described above, where they remained for 21 days before evaluation. The selected storage duration and atmosphere reflect standard commercial practice and allow consistent assessment of postharvest physiological responses across accessions.

#### 4.1.6. Equipment Calibration and Validation for Mechanical Resistance Tests

To ensure accuracy and consistency in the manual puncture tests, calibration and validation protocols were implemented for the handheld digital force gauge (ZTA 20N, IMADA, Toyohashi, Japan). Calibration was conducted prior to each experimental session using certified weights ranging from 0.1 N to 5 N. The gauge’s output was compared against these weights. Additionally, probe dimensions were verified using a micrometer with an accuracy of ±0.01 mm to ensure uniformity.

Validation was performed by testing a standard elastomeric material with known mechanical properties (Young’s modulus: 2 MPa). Measurements were repeated at three penetration depths (1 mm, 3 mm, and 5 mm) to simulate varying levels of fruit deformation. The coefficient of variation (CV) for repeated measurements was maintained below 5%, indicating high precision and reproducibility.

#### 4.1.7. Environmental Control Measures During Storage Experiments

Storage conditions were meticulously controlled to minimize variability and ensure reproducibility:

Storage temperature was maintained at 1.5 °C (±0.2 °C) using a programmable cold storage unit equipped with continuous monitoring. Temperature stability was verified at multiple locations within the chamber using calibrated digital thermometers with an accuracy of ±0.1 °C.

Uniform air circulation within the storage chamber was achieved using axial fans operating at a constant speed of 150 RPM. Airflow patterns were validated using an anemometer to ensure even distribution across all fruit samples.

#### 4.1.8. Fruit Damage Assessment

**Figure 8:**
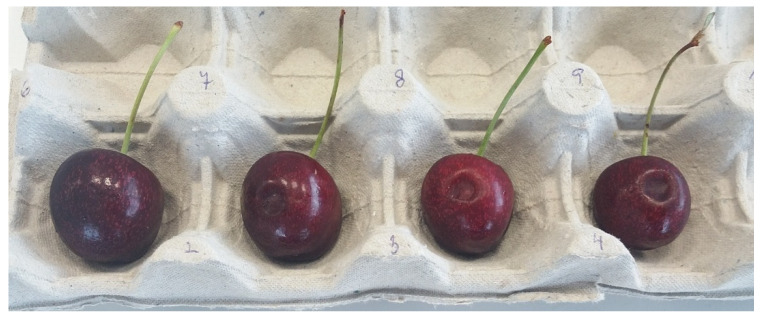
Classification of fruits into categories 1–4 (from left to right) according to the depth of surface pitting damage during the evaluation.

Following storage, the same 50 fruits per accession described in Section 4.1.4, Section 4.1.5 and Section 4.1.6. were visually examined and classified into four categories based on the severity of surface pitting damage, where the degree of damage was determined by the depth of the depressions using a 4-point scale as described in a previous study (Figure 8) [2]. No shelf-life period was applied; the fruits were evaluated immediately after removal from cold storage and were allowed to reach room temperature before damage assessment. Three technological replicates were included in the evaluation. The classification was as follows:

1. No damage (no visible depressions)
2. Slight damage (shallow depressions)
3. Moderate damage (moderate depressions)
4. Severe damage (deep depressions)

#### 4.1.9. Calculation of Damage Index

Based on this evaluation, a damage index (DI) scale was calculated, as described in a previous study [73], which served as the main indicator of susceptibility to surface pitting. The index was calculated as the sum of the number of fruits in each damage category (n1, n2, n3, n4) multiplied by one of 4 factors (1, 2, 3, 4), and the whole divided by the total number of fruits in the sample (N). The index was calculated using the following formula:

Damage Index (DI) = ((n1 × 1) + (n2 × 2) + (n3 × 3) + (n4 × 4))/N

### 4.2. Determination of Fruit Chemical Composition

Immediately after fruit picking, a 1000 g sample of stone free fresh fruits described in Section 4.1.4. was homogenized using a high-speed blender (Waring Commercial, Stamford, CT, USA) to ensure uniformity for chemical analysis. All measurements were performed in triplicate to ensure accuracy and reproducibility.

#### 4.2.1. Total Antioxidant Capacity (TEAC)

The total antioxidant capacity (TEAC) was assessed using the DPPH radical scavenging assay [74]. A 2 g aliquot of homogenized fruit was extracted with 10 mL of methanol (80%) in a centrifuge tube. The mixture was vortexed for 1 min and incubated at room temperature for 30 min in the dark. After centrifugation at 4000 rpm for 10 min, the supernatant was collected. A 200 µL aliquot of the extract was mixed with 800 µL of DPPH solution (0.1 mM in methanol) in a microplate well and incubated for 30 min in the dark. Absorbance was recorded at 515 nm using a UV-Vis spectrophotometer (Thermo Scientific, Waltham, MA, USA). The values were reported as µmol Trolox equivalents per 100 g fresh weight by comparing absorbance values to a standard curve prepared with Trolox solutions (0–200 µM). This extraction protocol primarily targets the readily extractable antioxidant compounds present in the cherry matrix and does not account for the bound antioxidant fraction.

#### 4.2.2. Anthocyanins

Anthocyanin content was assessed using high-performance liquid chromatography with diode-array detection (HPLC-DAD) [52]. Immediately after homogenization, the samples were kept protected from light and processed without delay to minimize enzymatic degradation of anthocyanins. A 5 g sample was processed with 15 mL of acidified methanol (0.1% formic acid) for extraction and sonicated for 15 min at room temperature. The extract was filtered through a 0.45 µm PTFE membrane filter before injection into an Agilent 1260 Infinity HPLC system equipped with a C18 column (150 × 4.6 mm, 5 µm particle size). The mobile phase was composed of solvent A (95% formic acid) and solvent B (90% methanol), applied in a gradient elution mode: 95% A at 0 min to 10% A at 10 min. Detection was conducted at 520 nm, and quantification was done using external standards as described below. The most abundant anthocyanins in cherry were quantified as cyanidin 3 glucoside and cyanidin 3 rutinoside. Anthocyanin standards (Extrasynthese, Genay, France) were stored at −18 °C and used for calibration. A stock solution of 1 mg/mL of each standard was prepared in 1% methanolic formic acid solution, from which working solutions were made at concentrations of 5, 10, 20, 50, 100, 150, and 250 µg/mL. The analyte contents of the samples were calculated using an external calibration curve and expressed as the amount of each anthocyanin per 100 g of fresh weight.

#### 4.2.3. Total Dry Matter (TDM)

Dry matter content was calculated gravimetrically by drying approximately 5 g of homogenized fruit in an aluminum dish at 105 °C until constant weight [59]. Dry matter content (%) was quantified as:

Total dry matter = (dry weight)/(fresh weight) × 100

This method ensures precision by accounting for all moisture loss during drying.

#### 4.2.4. Total Polyphenols

The total polyphenols were quantified using the Folin–Ciocalteu method [61]. A 1 g sample of homogenized fruit was extracted with 10 mL of methanol (80%) under continuous stirring for 1 h at ambient temperature. After centrifugation at 4000 rpm for 15 min, the supernatant was diluted tenfold with distilled water. A reaction mixture containing 200 µL of diluted extract, 1 mL of Folin–Ciocalteu reagent (diluted 1:10), and 800 µL of sodium carbonate solution (7.5%) was incubated for 30 min at ambient temperature in the dark. Absorbance was determined at 765 nm using a UV-Vis spectrophotometer, and results were presented as mg gallic acid equivalents per 100 g fresh weight. For each set of samples, a linear regression was constructed from the measured data in the form of the equation y = ax + b, in which y denotes absorbance and x denotes concentration (gallic acid equivalents). The quality of each curve was verified using the coefficient of determination (R^2^), which ranged from 0.9933 to 0.9985. This method ensures accurate calculation of polyphenol content by accounting for specific conditions of each measurement. It should be noted that this extraction procedure quantifies mainly the easily extractable polyphenols and does not include the fraction bound to cellular structures.

#### 4.2.5. Ascorbic Acid

Ascorbic acid concentration was determined using HPLC-DAD analysis with potassium dihydrogenphosphate as the mobile phase [67]. A homogenized sample (10 g) was weighed as rapidly as possible in an appropriate vessel and immediately extracted with metaphosphoric acid solution (20 g/L) to stabilize ascorbic acid and prevent enzymatic degradation in the lacerated tissues. The extract was filtered through a PTFE membrane filter (0.45 µm) before injection into an HPLC system equipped with a C18 column (250 × 4.6 mm, particle size: 5 µm). The mobile phase consisted of potassium dihydrogenphosphate buffer adjusted to pH 2.4 with phosphoric acid, delivered at a flow rate of 1 mL/min. Detection was carried out at 265 nm, and results were expressed as mg per 100 g fresh weight. Identification and quantification were carried out by comparison with an external calibration curve. A stock solution of L-ascorbic acid standard was prepared at a concentration of 1 g/L in the metaphosphoric acid extraction reagent, and working solutions were prepared at concentrations of 2, 5, 10, 20, 50, 100, and 200 mg/L by serial dilution. For each set of samples, a linear regression was constructed in the form of the equation y = ax + b, in which y denotes absorbance and x denotes concentration. The quality of each curve was verified using the coefficient of determination (R^2^), which ranged from 0.9991 to 1.00. Each sample was prepared in triplicate, and the reported values represent the mean of the three independent measurements.

### 4.3. Statistical Analysis

All statistical analyses were performed using Python 3.11.4 within the IDLE development environment, employing libraries such as NumPy, SciPy, pandas, and statsmodels. Data visualization was carried out using seaborn and matplotlib to ensure clear representation of results.

Descriptive statistics, including means, medians, standard deviations, minimum and maximum values, and quartiles, were calculated for all variables. The analysis was stratified by genotype, maturity stages, and year to summarize variability and data patterns.

To assess the effects of genotype, maturity stages, and fruit chemical composition on surface pitting resistance, a mixed linear model (LMM) was applied. The dependent variable was the damage index, determined from visual evaluations of surface pitting severity. Fixed effects included maturity stages (first or second), total antioxidant capacity (TEAC), anthocyanins, polyphenols, dry matter content, and ascorbic acid content. Random effects accounted for variability between genotypes and across years.

Model diagnostics were performed to validate the assumptions of the LMM:

Normality of residuals was assessed using the Shapiro–Wilk test.

Homoscedasticity (constant variance) was evaluated using Levene’s test.

Multicollinearity among predictors was examined using variance inflation factors (VIF).

Influential observations were detected using Cook’s distance.

Spearman’s rank correlation coefficients were computed to evaluate relationships between chemical parameters (e.g., TEAC, polyphenols) and the damage index. Correlation heatmaps indicating significance levels (*p* < 0.05) were created to illustrate these relationships.

Where appropriate, non-parametric tests were employed, with the Mann–Whitney U test comparing chemical composition between maturity stages.

Kruskal–Wallis tests with subsequent Bonferroni-corrected pairwise comparisons were used to investigate differences in surface pitting resistance indices among genotypes.

## 5. Conclusions

This study highlights the complex interactions between genetic, physiological, and environmental factors influencing cherry resistance to surface pitting. Genetic factors were the primary driver of surface pitting resistance. Physiological traits, particularly dry matter content, determined resistance. Genetic control over resistance was strong and consistent across seasons. Accession ‘10072’ demonstrated superior and stable resistance across seasons, highlighting the potential for genetic selection.

These findings provide breeders and growers with useful criteria—such as dry matter content and ascorbic acid levels—for rapid screening of accessions with better storability, and they underline the potential of integrating genetic selection with physiological markers to improve postharvest performance Future research should focus on identifying genetic markers and exploring epigenetic regulation of antioxidant pathways to further enhance our understanding of resistance mechanisms and support precision breeding.

## Figures and Tables

**Table 1:** Analysis comparing principal physiological and mechanical properties of sweet cherry (*Prunus avium* L.) fruits across two maturity stages. The table summarizes mean values and standard deviations (SD) for Total Antioxidant Capacity, Total Anthocyanins, Total Dry Matter, Total Polyphenols, Ascorbic Acid, and Damage Index. Differences between the two maturity stages were tested for statistical significance using the Mann–Whitney test, with *p*-values reported for each parameter (*p* < 0.05 denotes significant variation).

Variable	1st Harvest	SD	2nd Harvest	SD	*p* Value
Total Antioxidant Capacity (µmol Trolox/100 g)	106.70	25.57	105.88	31.09	>0.05
Total Anthocyanins (mg/100 g)	3.88	5.04	9.48	11.16	<0.001
Total Dry Matter (%)	12.23	2.58	13.73	3.00	<0.001
Total Polyphenols (mg GAE/100 g)	28.93	14.83	29.48	11.28	>0.05
Ascorbic Acid (mg/100 g)	6.04	1.85	6.86	2.09	<0.001
Damage Index	2.17	0.55	2.19	0.52	>0.05

**Table 2:** Spearman’s correlation coefficients along with *p*-values (*p* < 0.001) are displayed in the correlation matrix for relationships between total antioxidant capacity, anthocyanins, dry matter, total polyphenols, ascorbic acid, and damage index at the second maturity stage.

Variable	Total Antioxidant Capacity	Total Anthocyanins	Total Dry Matter	Total Polyphenols	Ascorbic Acid
Total Antioxidant Capacity	1	−0.323	−0.483	0.019	−0.058
Total Anthocyanins	−0.323	1	0.554	0.389	0.228
Total Dry Matter	−0.483	0.554	1	0.316	0.260
Total Polyphenols	0.019	0.389	0.316	1	0.090
Ascorbic Acid	−0.058	0.228	0.260	0.090	1
Damage index	0.309	−0.187	−0.445	−0.086	−0.148

**Table 3:** Comparison of key physiological and mechanical characteristics in sweet cherry (*Prunus avium* L.) fruits over two years. The table summarizes mean values and standard deviations (SD) for Total Antioxidant Capacity, Total Anthocyanins, Total Dry Matter, Total Polyphenols, Ascorbic Acid, and Damage Index. Statistical significance between maturity stages was evaluated using appropriate tests, with *p* values provided for each parameter (*p* < 0.05 indicates significant differences).

Variable	1st Year	SD	2nd Year	SD	*p* Value
Total Antioxidant Capacity (µmol Trolox/100 g)	88.92	26.23	123.92	18.02	<0.001
Total Anthocyanins (mg/100 g)	12.00	11.34	2.45	3.25	<0.001
Total Dry Matter (%)	15.52	2.08	10.99	1.61	<0.001
Total Polyphenols (mg GAE/100 g)	33.37	11.84	24.85	12.98	<0.001
Ascorbic Acid (mg/100 g)	6.62	1.95	6.30	2.07	>0.05
Damage Index	1.95	0.43	2.41	0.54	<0.001

## Data Availability

The original contributions presented in this study are included in the article. Further inquiries can be directed to the corresponding author.
